# The Variation of SARS-CoV-2 and Advanced Research on Current Vaccines

**DOI:** 10.3389/fmed.2021.806641

**Published:** 2022-01-18

**Authors:** Yao Jiang, Qian Wu, Peipei Song, Chongge You

**Affiliations:** Laboratory Medicine Center, Lanzhou University Second Hospital, Lanzhou, China

**Keywords:** SARS-CoV-2, variants, mutations, transmissibility, immune responses, vaccines

## Abstract

Over the past 2 years, the severe acute respiratory syndrome coronavirus 2 (SARS-CoV-2) caused the coronavirus disease 2019 (COVID-19) and rapidly spread worldwide. In the process of evolution, new mutations of SARS-CoV-2 began to appear to be more adaptable to the diverse changes of various cellular environments and hosts. Generally, the emerging SARS-CoV-2 variants are characterized by high infectivity, augmented virulence, and fast transmissibility, posing a serious threat to the prevention and control of the global epidemic. At present, there is a paucity of effective measurements to cure COVID-19. It is extremely crucial to develop vaccines against SARS-CoV-2 and emerging variants to enhance individual immunity, but it is not yet known whether they are approved by the authority. Therefore, we systematically reviewed the main characteristics of the emerging various variants of SARS-CoV-2, including their distribution, mutations, transmissibility, severity, and susceptibility to immune responses, especially the Delta variant and the new emerging Omicron variant. Furthermore, we overviewed the suitable crowd, the efficacy, and adverse events (AEs) of current vaccines.

## Introduction

**Figure 1:**
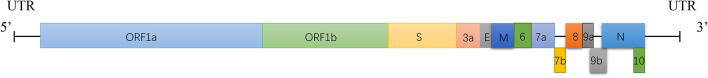
The genome structure of SARS-CoV-2 viruses. The whole length of SARS-CoV-2 genome is nearly 30 kb, with 11 ORFs and encoding 27 viral proteins. In general, SARS-CoV-2 genome is capped at the 5'UTR and polyadenylated at the 3'UTR. The S, E, M, and N genes encode structural proteins. While, ORF1a and ORF1b, occupying approximately the two-thirds of full-length genome, belong to the genes that encode non-structural proteins, containing nsp1 to nsp16. Also, the rest of the genes encode the accessory proteins. UTR, untranslated regions; ORFs, open reading frames; S, spike; E, envelope; M, membrane; N, nucleocapsid.

The coronavirus disease 2019 (COVID-19) spreads widely, caused by severe acute respiratory syndrome coronavirus 2 (SARS-CoV-2), which led to transmissible acute respiratory contagious diseases worldwide (1), arousing public concern and global health issues. SARS-CoV-2 exhibited ~79% identity with SARS-CoV and 50% with the Middle East Respiratory Syndrome (MERS) coronavirus (MERS-CoV) (2). It is a class of RNA viruses, belonging to the lineage of B βCoV, characterized by positive-sense, single-stranded, and enveloped traits, with the length of 29,903 nucleotides, 11 open reading frames (ORFs), and encoding 27 viral proteins. The ORF1a/b is marked by 21,290 nucleotides in length and encodes 16 non-structural proteins, namely nsp1 to nsp16. The last part of the structure of the SARS-CoV-2 genome contains a total of 8,613 nucleotides, which encodes four structural proteins and some accessory proteins. The structural proteins are spike (S), envelope (E), membrane (M), and nucleocapsid (N) proteins; the accessory proteins are an array of ORFs, that is, ORF 3, 6, 7a, 7b, 8, and 10 (3). Figure 1 shows the genome structure of SARS-CoV-2 viruses from 5'UTR to 3'UTR, covering the regions of ORF1a/b, S, E, M, N, as well as ORF 3, 6, 7a, 7b, 8, 9a, 9b, and 10.

It is well-known that mutations easily occur in the gene sequence of viruses, where SARS-CoV-2 is no exception (4). Compared to DNA viruses, RNA viruses are less stable for their single-stranded structure, which is easy to result in fracture and recombination. Also, due to the instability and low enzyme activity of RNA to repair the error during the replication process, the RNA virus easily mutates. Accordingly, RNA viruses are more likely to cause diseases and are deadly to the hosts. Similar to most RNA viruses, SARS-CoV-2 tends to evolve into all sorts of novel variants during transmissible progression. Due to the flexible adaptability of SARS-CoV-2 in various cellular environments and diverse hosts, it has rapidly spread among the population during the COVID-19 pandemic. Meanwhile, genetic sequence mutations also help SARS-CoV-2 to spread quickly and aggravate conditions (5, 6). Scientists have found that the S gene possesses an ability to mutate into a more infectious form, which has been regarded as a clinical variant of concerns (VOCs) and appears to be the cause of increased transmissibility and immune escape for antibodies. Besides, the WHO formally explained that SARS-CoV-2 VOCs are endowed with higher contagiousness, quick transmissibility, augmented virulence, and are insensitive to vaccines (7).

The S glycoprotein is essential for SARS-CoV-2 to promote combination with receptors and entry into cells (8). The N-terminal S1 subunit, mediating bonding with angiotensin-converting enzyme 2 (ACE2) receptors and C-terminal S2 subunit, which is responsible for cellular fusion, is incorporated into the S glycoprotein (9). Besides, Zhan et al. demonstrated that the appearance of various unique alleles in S genes than the E, M, N genes in 3,090 isolates from plenty of countries, implies that the existence of a large amount of genetic diversity in S gene might be beneficial for viral survival (10). Since the S glycoprotein of SARS-CoV-2 is capable of entering into cells by integrating with ACE2 receptors, once mutations occur in S genes, the affinity of receptor and immunogenicity of viruses will be altered, and an immune escape will easily occur (11). To our knowledge, S glycoprotein alterations in all the variants of SARS-CoV-2 nearly shared D614G mutation, a nonsynonymous mutation causing aspartic acid at position 614 to change to glycine (D614G) (12) among various variants. D614G mutation endowed SARS-CoV-2 to open more than two receptor-binding domains (RBDs), higher than wild-type D614 protein that opens merely one RBD (13–15), making it possible for SARS-CoV-2 to rapidly spread (16). Some researchers (17, 18) demonstrated that D614G mutation was able to promote the entry of SARS-CoV-2 into cells and membrane fusion, leading to quick transmissibility of viruses. Besides, G614 mutation with smaller CT values *via* RT-qPCR detection may be germane to higher SARS-CoV-2 viral loads in the upper respiratory tract of patients (19), but irrelevant to severity and mortality of COVID-19 (12).

A large quantity of SARS-CoV-2 variants have been recorded during the period of COVID-19 pandemic (20). For SARS-CoV-2 viruses, the basic reproduction (R0: the estimated numbers of susceptible individuals with secondary infections transmitted by infected subjects) was about 2.50 during the pandemic period in Wuhan but now the R0 has reached as high as 6.10 for the various variants (21, 22). Of note, five significant variants aroused public extensive attention, including Alpha (B.1.1.7, Q.1-Q.8) (23), Beta (B.1.351, B.1.351.2, B.1.351.3) (24), Gamma (P.1, P.1.1, P.1.2) (25), Delta (B.1.617.2 and AY.1 sublineages) (26), and Lambda (C.37) (27) variants. Currently, up to September 22, 2021, according to the latest report released by the US government's SARS-CoV-2 Interagency Group (SIG) [available from: SARS-CoV-2 Variant Classifications and Definitions (cdc.gov)], it was defined that among a total of four categories of SARS-CoV-2 variants, that is, VBM, including Alpha, Beta, B.1.617.3., Gamma, Epsilon (B.1.427 and B.1.429), Eta (B.1.525), Iota (B.1.526), Kappa (B.1.617.1), Mu (B.1.621, B.1.621.1), and Zeta (P.2) variants, VOC, only the Delta variant contained, variant of interest (VOI), and variant of high consequence (VOHM), where no SARS-CoV-2 variants has been designated as VOI and VOHM groups. In contrast, till September 22, 2021, WHO has still designated the Alpha, Beta, Gamma, and Delta variants as VOC. At the same time, the Lambda is defined as VOI by WHO [available from: Tracking SARS-CoV-2 variants (who.int)]. However, on November 26, 2021, WHO has designated a new emerging B.1.1.529 variant as a VOC, named Omicron [available from: Classification of Omicron (B.1.1.529): SARS-CoV-2 Variant of Concern (who.int)].

For investigating the influence of various emerging variants on public health, and making more effective strategies to battle against SARS-CoV-2 and variants, the study aims to summarize the emerging SARS-CoV-2 variants and their main features, as well as other current advanced studies on vaccines against them worldwide. Table 1 summarizes the characteristics of the main variants of SARS-CoV-2 viruses, including the information of next strain clade, places first identified, mutations in S protein, as well as their transmissibility, infectivity, immune escape, etc.

**Table 1:** The characteristics of main variants of SARS-CoV-2 viruses.

**Variants**	**Nextstrain clade**	**First identified**	**Spike protein mutations**	**Characteristics**
Alpha	20I/501Y.V1	UK	ΔH69, ΔV70, Δ144, (E484K*), (S494P*), N501Y, A570D, **D614G**, P681H, T716I, S982A, D1118H (K1191N*)	Rapid transmissibility and higher infectivity
Beta	20H/501.V2	South Africa	D80A, D215G, Δ241, Δ242, Δ243, V367F, P384L, R408I, K417N, E484K, N501Y, **D614G**, A701V	Higher viral infectivity and immune escape
Gamma	20J/501Y.V3	Japan/Brazil	L18F, T20N, P26S, D138Y, R190S, K417T, E484K, N501Y, **D614G**, H655Y, T1027I, V1176F	Augment of viral transmissibility
Delta	21A/S:478K	India	T19R, (V70F*), T95I, G142D, E156-, F157-, R158G, (A222V*), (W258L*), (K417N*), L452R, T478K, **D614G**, P681R, D950N	Most contagious; higher viral replication; and leading to severe illness
Delta plus	NA	India	T95I, G142D, R158G, L452R, T478K, K417N	Increased transmissibility; high affinity with pulmonary epithelial cells; and immune evasion
Omicron	21K	South Africa	A67V, ΔH69, ΔV70, T95I, G142D, ΔV143, ΔY144, ΔY145, ΔN211, L212I, ins214EPE, G339D, S371L, S373P, S375F, K417N, N440K, G446S, S477N, T478K, E484A, Q493R, G496S, Q498R, N501Y, Y505H, T547K, **D614G**, H655Y, N679K, P681H, N764K, D796Y, Q954H, N969K, L981F, and N856K	Increased viral replication, infectivity and re-infection; increased transmissibility; immune escape; recombination with HCoV-229E viruses

UK, United Kingdom; Δ, deletion; ins, insertion; *, detected in some sequences but not all; NA, not available. The bold values show the shared mutation in all variants.

## Predominant Variations of SARS-CoV-2

**Figure 2:**
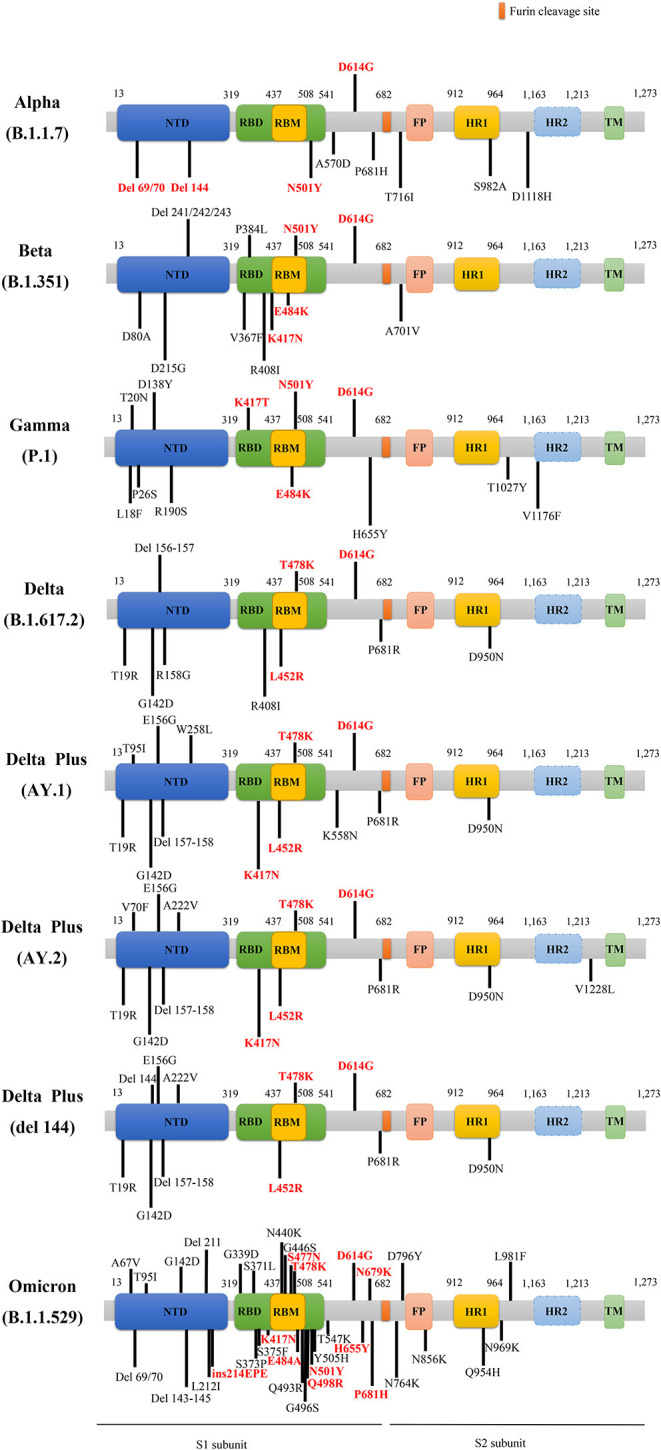
The schematic diagram of the corresponding mutation sites of predominant variants in S protein. The red D614G mutation is shared by these main variants. The rest of the mutations labeled red belong to key mutations in the respective variants. NTD, N-terminal domain; RBD, receptor binding domain; RBM, receptor-binding motif; FP, fusion peptide; HR1, heptad repeat 1; HR2, heptad repeat 2; TM, transmembrane region; del, deletion; ins, insertion.

There are several main variants of SARS-CoV-2, including the Alpha, Beta, Gamma, Delta, Delta plus, and Omicron variants. Figure 2 presents a schematic diagram of the corresponding mutation sites of predominant variants in the S protein.

### Alpha (B.1.1.7 Lineage) Variant

The SARS-CoV-2 variant of B.1.1.7 lineage, also called 20I/501Y.V1 or VOC 202012/01, was recently named as an Alpha variant by the WHO. It was first discovered in Kent, UK, becoming the predominant strain of the United Kingdom and gradually prevailing around Europe (28). The reproduction number in the Alpha lineage was higher by 43–90% than the preexisting variants, and the transmission increased in three countries, including Denmark, Switzerland, and the United States, fluctuating between 59 and 74% (29). Compared to the original strains, two-thirds of higher death cases were observed in patients with the Alpha variant infection in the UK (30). Up to March 29, 2021, the Alpha variant accounted for nearly 95% of SARS-CoV-2 infections in the UK and has caused dissemination in 114 countries (31). Besides, Cetin et al. (2021) collected the data of 3,700 COVID-19 patients from April 2020 to March 2021 in Tokat, Turkey, of which 30% were infected with the Alpha variant, increasing the local hospitalization rate (32).

It was reported that the Alpha lineage was characterized by 23 genetic mutations in comparison with previous SARS-CoV-2 strains, and the Alpha variant carried other eight mutations in the S gene, containing del H69/V70 (ΔH69/V70), del Y144 (ΔY144), N501Y, A570D, P681H, T716I, S982A, and D1118H, besides the D614G mutation (33) (Figure 2). Especially, the mutations of ΔH69/V70, ΔY144, and N501Y endowed SARS-CoV-2 variant with rapid transmissibility and high infectivity (34). Spontaneous deletion of H69/V70 was defined as a double deletion of histidine 69 and valine 70 at the NTD site (33), which was related to immune evasion in patients with hypoimmunity and enhancement of viral infectivity (29). Furthermore, researchers found that a synergy between ΔH69/V70 and D614G or N439K mutations, caused immune escape and infectivity augment (35, 36). The Alpha variant can be effectively neutralized by vaccines that target RBD-specific regions but a loss of efficacy for NTD-specific antibodies may be ascribed to ΔY144 that participates in neutralization evasion since ΔH69/V70 alteration alone cannot make it resistive to antibodies (37). The N501Y mutation referred to as tyrosine (Y) replacement for asparagine (N) at the 501 site, is located in the receptor-binding motif (RBM) region of S gene, and promoted the affinity of the variant with ACE2 receptors (38–40), enhancing the viral adherence and its subsequent entry into the host cells. Meanwhile, the Alpha variant harboring N501Y mutation reduced nearly 9% of affinities apparently to those neutralizing antibodies than the wild type (WT) (41).

Besides, E484K mutation, vital for immune escape, has been substantiated that it exerted a crucial impact on the neutralization of antibodies (42, 43), particularly the simultaneous incidence of N501Y and K417N mutations (44). SARS-CoV-2 variants, with the combination of E484K, K417N, and N501Y mutations made it challenging to perform antibody treatment and abated the potency of corresponding vaccines (45). Notably, P681H mutation was proline (P) to histidine (H) mutation in the furin cleavage position of S protein, then it influenced the conformational stability of S protein, resulting in the increase of infectivity in the Alpha variant (46). Based on extensive researches, the current vaccines for SARS-CoV-2 are effective and their efficacy against the Alpha variant is unimpaired (43, 47, 48).

### Beta (B.1.351 Lineage) Variant

The emergent SARS-CoV-2 variant of B.1.351 lineage (also called 501Y.V2), renamed as the Beta variant by WHO, was first found in early October 2020, in South Africa (49), triggering the second wave of SARS-CoV-2 infection. Thus, the Beta variant spread rapidly across many countries (50) and was detectable soon (24, 51, 52). Pearson et al. forecast the transmissible speed of the Beta strains, nearly 1.5 times (95% CI: 1.20–2.13) than the previous circulating SARS-CoV-2 strains (53). The Beta variant caused reinfection for patients infected with COVID-19, and also contributed to infection among healthy subjects who were inoculated with the first dose of ChAdOx1 vaccine, a recombinant adenoviral vector targeting the S protein of SARS-CoV-2, in Dhaka, Bangladesh (54). At present, there are insufficient pieces of evidence for the impact of the Beta variant based on the severity of the condition.

The mutations of the Beta variant are shown in Figure 2, covering the main K417N, E484K, N501Y, and D614G mutations, as well as other mutations, such as D80A, D215G, del 241 (Δ241), del 242 (Δ242), del 243 (Δ243), V367F, P384L, R408I, D6101G, and A5101V. The Beta variant harboring N501Y, K417N, and E484K mutations caused resistance to the antibody therapy (52, 55), among which E484K mutation was able to decrease the variants' susceptibility to the potency of antibodies (44), triggering immune escape (Figure 2). Besides, K417N and E484K mutations were able to induce conformational alterations of S protein, which were crucial for binding to ACE2 receptors and participating in the recognition of antibodies (56), consequently resulting in the enhancement of viral infectivity (57). It is worth noting that the nucleotide substitution of G23012A in the Beta variant participated in the E484K mutation, considered to involve in altering viral antigenicity and in turn causing low efficacy of vaccines (44, 47, 48). Although there is resistance to antibodies neutralization in the Alpha and Beta variants, vaccines can still generate protection against SARS-CoV-2 to a certain degree in the population who receive vaccination (58–61).

### Gamma (P.1 Lineage) Variant

The B.1.1.28.1 linage, known as P.1, 20J/501Y.V3 or Gamma variant, was first detected in four travelers who took a trip from Brazil to Tokyo, during a routine screening in the airport of Tokyo, Japan, in January 2021 (62, 63). Viral loads were nearly ten folds higher in the Gamma variant infections than in the non-P.1 strains (64); hence patients infected with the Gamma variant are more contagious (65, 66). Until February 2021, more than 51.1% of cases suffered from SARS-CoV-2 P.1 variant in all COVID-19 patients who were identified in Umbria, Italy (67). And the Gamma variant began to emerge in more than 45 countries until March 30, 2021, according to the release of the epidemiological information by WHO, including the United States (68), Spain (69), Bangladesh (70), Uruguay (71), Italy (72), etc.

The Gamma variant had 12 mutations in S Protein, namely L18F, T20N, P26S, D138Y, R190S, K417T, E484K, N501Y, D614G, H655Y, T1027I, and V1176F substitutions (62) (Figure 2). The mutations of K417T, E484K, and N501Y might facilitate affinity with ACE2 receptors and immune escape (73, 74). The increasing affinity of the Gamma variant binding to ACE2 to some extent is equal to the Beta but stronger than the Alpha, which rendered the augmentation of viral transmissibility and enabled them to become primary strains in the areas where they arrived (75, 76). Remarkably, the Gamma appeared to be not as resistant as the Beta but similar to the Alpha in antibody responses that were acquired naturally or generated by vaccines (75), owing to RBD alterations that influence antibodies neutralization (73). The antibodies produced by convalescent patients previously infected with original SARS-CoV-2 cannot effectively neutralize the Alpha and Gamma variants, but antibodies induced by vaccines can defend against these strains up to a point (77–79).

### Delta (B.1.617.2 Lineage) Variant

The most concerned SARS-CoV-2 mutant is the B.1.617.2 that was first uncovered in India (80), and then, it was named as the Delta variant by the WHO. The Delta variant was the prime cause of the second wave of fatal COVID-19 infection in India in April 2021. Before that, the variant was first detected in March 2021 in the USA; then the Delta variant raised to prevalent strains in the next several weeks. In addition, the genome sequencing of the Nanjing COVID-19 cases showed that the origin of the SARS-CoV-2 virus was a Delta variant. By spreading to multitudes of countries around the world, the Delta variant has gradually become a global epidemic strain. The Delta variant carried double mutations (L452R and E484Q) in S protein (81, 82), which caused its stronger transmission, higher viral loads, shorter infection incubation, a longer period of viral shedding in pharyngeal swab specimens, and higher danger of exacerbation to critical status in patients infected with SARS-CoV-2 viruses. Also, the Delta variant presented a lower speed of viral clearance in comparison with WT strains (83). Meanwhile, the Delta plus variant (also called AY.1 or B.1.617.2.1) also deserves public attention. The Delta plus variant further underwent evolution but not as a simple mutation (K417N) of the Delta variant, which was first found in India and spread to a multitude of countries (84). The three worrying hallmarks of the Delta plus variant include increased transmissibility, powerful combination with receptors of pulmonary epithelial cells, and abatement of interaction with monoclonal antibodies [available from: Delta plus Covid variant: Here's what you need to know (cnbc.com)].

Centers for Disease and Control Prevention (CDC) proposed that the R0 of the Delta variant fluctuates from 5 to 9.5 (85), with transmissibility faster than MERS and SARS, smallpox, common cold, Spanish flu, and Ebola. The Delta variant now is more transmissible 1.1–1.4-fold than the previous strains (86). Meanwhile, it is possible for the Delta variant to spread so fast, which is mainly attributed to viral loads in patients infected with the Delta variant being roughly 1,000 times more than the original strains (87). It is reported that the Delta variant is adapting more to human bronchial epithelial cells, has significantly higher viral replication and easier transmissibility (86), and has a 60% more increased risk rate in the aspect of household transmission than the Alpha variant (88).

Indeed, the Delta variant was capable of causing more serious illness than the Alpha variant or ancestral strains. In Canada, the Delta strains brought about higher hospitalization [adjusted odds ratio (aOR): 2.08, 95%CI: 1.80–2.38)], ICU admission (aOR: 3.34, 95%CI: 2.64–4.31), and death (aOR: 2.32, 95%CI: 1.47–3.30) (89). And in Singapore, the Delta variant was pertinent to the higher oxygen demand, ICU admission, or death (aOR: 4·90, 95% CI: 1.43–30.78), as well as pneumonia (aOR: 1.88, 95% CI: 0.95–3.76) (90). While, in Scotland, the risk of hospitalization driven by the Delta VOC also escalated, with a hazard ratio (HR) of 1.85 (95% CI: 1.39–2.47) (91). Therefore, the Delta variant can cause a higher hospitalization rate than the Alpha variant or original viruses (89, 91).

Many pieces of evidence show that the Delta variant can lead to the appearance of more severe diseases than ancestral viruses among unvaccinated subjects (90). First, the infection rate of the Delta variant significantly increased in the young population who are not inoculated with vaccines (92). In the earlier local transmission of the Delta variant in Guangzhou, a large number of infected cases was young individuals aged <18 years who achieved quick transmissibility by three ways, namely short-distance touch, household, and community spread (83). Second, the Delta variant is more liable to infect and transmit viruses among those who were unvaccinated, which is the potential risk of widespread transmission. Third, the amount of Delta variant viruses in fully immunized individuals goes down quicker than that in the unvaccinated population (93). Nevertheless, what is worrying is that the vaccinated rate of the population from numerous Asian countries seems to be far from enough (82). Owing to uneven vaccination status in some developing countries, such as Japan, Indonesia, Iraq, and Vietnam (82), the Delta viruses confronted immune pressure to some degree, whereas this insufficient immunization enabled the variants to be more transmissible. Also, the low coverage of vaccination in numerous communities drives rapid and dramatic emergence of cases infected with the Delta variant (86), which may increase the opportunities for viral variation.

As is known, the S protein can interact with ACE2 and TMPRSS2 (58), then mediate cellular entry and affect the viral infectivity. It is reported that the Delta lineage harbors several mutations in S protein, containing distinctive mutations, namely T19R, del 157/158 (Δ157–158), L452R, T478K, D614G, P681R, D950N (94), and other mutations, i.e., G142D, T95I, and R158G (Figure 2) (95). Intriguingly, L452R and T478K, the specific mutations of the Delta variant (81), as well as deletions at the N-terminal region, may exert influence on immune responses targeting crucial antigen site of receptors (96). Especially, L452R and T478K mutations obviously stabilized the structure of RBD-ACE2 complex. Besides, P681R mutation occurs in the cleavage site of the S1-S2 subunits, promoting interactions with furin, driving membrane fusion, facilitating viral transmissibility (81), and increasing the virus replication thus contributing to higher SARS-CoV2 viral loads (97).

Besides, compared to the Delta variant, the Delta plus variant possesses six crucial mutations, namely T95I, G142D, R158G, L452R, T478K, and K417N, rather than only owning a K417N mutation. Figure 2 depicts the main mutations in S protein of the Delta plus variants, including the delta AY.1, delta AY.2, and delta Δ144 (98). It is noteworthy that the K417N mutation seems to cause immune evasion by losing K417 interaction with Y52, thus reducing antibodies binding to S protein (84).

Some studies revealed that the vaccine efficacy (VE) modestly reduced with the extension of inoculation time, especially in the aged adults. The VE against the Delta variant in England and the United States was 88% (99) and 66% (100), respectively, but an apprehensive report by the Israeli Health Ministry suggested that Pfizer mRNA vaccines had only 39% protection against symptomatic COVID-19 disease caused by the Delta variant in Israel on July 23, 2021. In essence, some research also elucidated that neutralization of antibodies from the serum of convalescent COVID-19 and antibodies induced by Covaxin vaccines against the Delta variant showed a reduction of 4.6 and 2.7 times, respectively (101). However, Pfizer-BioNTech BNT162b2 or AstraZeneca-Oxford ChAdOx1 nCoV-19 vaccines, are also capable of defending against the Delta variant (85, 99, 102, 103), lowering 50–60% the infection rate of the Delta variant, once the subjects received two doses of vaccines (102). Additionally, it was observed that Delta plus, Lambda, and other variants failed to escape neutralization of vaccine-elicited antibodies although the presence of antigenic alterations was observed (98). Consequently, it is imperative to popularize vaccines worldwide to effectively prohibit public infection with various SARS-CoV-2 variants.

### Omicron (B.1.1.529 Lineage) Variant

On November 24, 2021, a novel B.1.1.529 variant has emerged and was first reported in South Africa. It was designated as a VOC and named Omicron variant by the WHO on November 26, 2021 [available from: Classification of Omicron (B.1.1.529): SARS-CoV-2 Variant of Concern (who.int)]. The epidemiological report indicated that infections caused by B.1.1.529 variant in reported cases have increased sharply in the recent weeks. Based on a recent study, researchers described the epidemiology of Omicron strain and pointed out that the Omicron variant was unable to increase and even reduced the risk of primary infection in the population, but it increased the hazard of reinfection. Furthermore, in the second and third waves of SARS-CoV-2 infections dominated by Beta and Delta strains, the HR of reinfections were found to be 0.75 (95%CI: 0.59–0.97) and 0.71 (95%CI: 0.56–0.92), respectively. In contrast, for wave 4 SARS-CoV-2 infections dominated by the Omicron variant in South Africa, the HR of reinfections rose to 2.39 (95%CI: 1.88–3.11) (104). In addition, researchers discovered roughly 37 predominant alterations to S protein in the Omicron variant (105) (Figure 2, not yet peer-reviewed), where K417N, S477N, Q498R, E484A, and N501Y mutations can help viruses dodge antibody neutralization; thus, the immune escape caused by the Omicron variant may be more obvious, while some mutations occurring in the Furin site, namely H655Y, N679K, and P681H, allow for the entry of viruses into cells and enhance the viral replication and infectivity [available from: Omicron variant may spread more easily than other COVID versions because it contains a piece of common cold virus, scientists say (granthshala.com)]. Of note, the insertion mutation (ins214EPE) that was expressed in seasonal coronaviruses (e.g., HCoV-229E) was first identified in the Omicron variant without being found in any SARS-CoV-2 strains previously. Single cell RNA-sequencing indicated that the host's respiratory and gastrointestinal cells coexisted with the Omicron strain and HCoV-229E, which might create the conditions for the recombination of these two viruses [available from: OSF Preprints | Omicron variant of SARS-CoV-2 harbors a unique insertion mutation of putative viral or human genomic origin]. Although the Omicron variant is characterized by the potentiality of super strains, it deserves not to be panicking at present and it is recommended to accelerate vaccination in the weak areas of the world as soon as possible for the prevention of the reemergence of “super strains” [available from: Heavily mutated Omicron variant puts scientists on alert (nature.com)].

## Other SARS-CoV-2 Variants

The VOIs have caused COVID-19 clusters or considerable community transmission, with a gradual growing prevalence over time, posing an emerging threat to public health (106). Up to September 22, 2021, according to the newest classification by the WHO [available from: Tracking SARS-CoV-2 variants (who.int)], two SARS-CoV-2 variants are defined as VOIs, namely Lambda (also known as B.1.1.1 or C.37 lineage) and Mu (B.1.621 lineage). Also, it is reported that the first patients with the Lambda variant dated to August 2020, in Peru, according to a weekly epidemiological report from the WHO (107). The data from WHO implied that 81% of new cases in Peru derived from Lambda variant infections during April–June 2021 (107, 108), and the per-capita mortality rate of COVID-19 soared to the highest in the world. And there is an expanding trend for the Lambda variant and it has been detected in many countries (109). There are several mutations, mainly containing G75V, T76I, del 246–252 (Δ246–252), L452Q, F490S, D614G, and T859N. Two novel mutations of Lambda variant, such as L452Q and F490S, enabled the Lambda strains to be resistant to antibodies neutralization. Of note, when the L452Q mutation enhanced the affinity of Lambda variant with ACE2 receptors, then infectivity increased roughly to two-fold (110).

The Mu variant was first detected and became sporadic in Colombia in January 2021 [available from: SARS-CoV-2 variants of concern as of 16 September 2021 (europa.eu)]. It belongs to B.1 lineage descendants with high mutations, encompassing the insertion of 146 N (ins 146N) and mutations in S protein (Y144T, Y145S, R346K, E484K, N501Y, and P681H). To date, the Mu variant travels across nearly eight countries, containing Colombia, United States, Spain, Netherlands, Denmark, Mexico, Germany, and Curacao. The enhancement of SARS-COV-2 genomic surveillance for the third peak of the COVID-19 pandemic in Colombia is responsible for the high frequent emergence of the Mu variant (111). However, the neutralization of serum antibodies derived from individuals vaccinated with two doses of BNT162b2 vaccine to the Mu variant is still robust and potent, even though the neutralization is lower than other B.1 lineages of SARS-CoV-2 (112).

Recently, the WHO proposed a percept of variants under monitoring (VUM), referring to variants with several inheritable changes that are speculated to influence viral traits and may pose a threat to the public in the future. Nevertheless, since there is an absence of adequate evidence on phenotypic or epidemiological markers, it is needed to be monitored and evaluated based on the pending novel evidence [available from: Tracking SARS-CoV-2 variants (who.int)]. Here we discuss four VUMs, including Kappa (113), Iota (114), Eta (115), and Epsilon variants.

Both the Kappa variant, which is first identified in India in December 2021, and the Delta variant belong to one of the subspecies of Indian strains. The mutations (L452R, T478K, E484Q, D614G, and P681R) in S protein make the Kappa variant, similar to the Delta virus, but more susceptible to infect cells and the escape antibodies response of the immune system (81). Owing to the Kappa variant attenuating susceptibility to neutralization of antibodies, this variant more readily lowers the VE and increases the chance of reinfection (116). Nevertheless, studies indicated that RBD immune sera (117) and BNT162b2 induced sera (118), or mRNA-1273 elicited sera (113) were still efficacious against the Kappa variant, although both L452R and E484Q mutations (119) drive the Kappa variant to become resistant to antibodies neutralization (120–122).

Eta and Iota (123) variants were first uncovered in New York, in November 2020 (38). The Eta variant carrying a single mutation (either N501Y or E484K) increased the affinity of RBD with ACE2 receptors (124), thus resulting in a conformational change that made it possible for the Eta variant to be better neutralized by antibodies *via* exposure of the functional epitope (79). Besides, it was reported that the transmissibility and fatality caused by the Iota variant remarkably improved. Compared to other variants, from November 2020 to April 2021, the mortality of people aged 45–65, 65–74, and more than 75, who were infected by the Iota variant, increased to 46, 82, and 62%, respectively (125). The E484K mutation enables the Iota variant to spread and rise sharply, replacing previous epidemic variants in New York (123). Whereas, preliminary clinical data implied that the Iota variant, even though it harbors E484K mutation, was unable to cause severe illness (114).

The Epsilon (B.1.427/B.1.429) variant first occurred in Los Angeles County in July 2020, and subsequently began to prevail in California on January 22, 2021 (126). At first, the proportion of Epsilon variants carrying L452R mutation was 24.8% of all COVID-19 cases in December 2020; thereafter, the incidence of this variant increased to 62.5% in March 2021 (127). Besides, the viral loads of the Epsilon variants derived from swab samples are approximately higher by two-fold than the non-Epsilon strains, with an underlying increase in the transmissibility of viruses. Meanwhile, the Epsilon variants were found to be resistant to neutralization of antibodies from convalescent patients and vaccine recipients *in vitro* (128). A study delineated that the Epsilon variants reduced the susceptibility to antibodies induced by Moderna mRNA1273 and BNT162b2 vaccines, mainly ascribing to the appearance of S13I and W152C mutations that resulted in immune escape (129).

## Current Vaccines

Given that there are no specific drugs to cure COVID-19, it is extremely imperative to establish an effective immunologic barrier of the population *via* vaccination to fight against various SARS-CoV-2 and variants. Here, we summarized the best clinical and therapeutic approaches for COVID-19 illness (Table 2). Recently, some vaccines have been developed against SARS-CoV-2 and their variants, encompassing Pfizer/BioNTech mRNA vaccine, Moderna mRNA vaccine, AstraZeneca-Oxford vaccine, CoronaVac, DNA vaccine, Beijing Institute of Biological Products inactivated vaccine (BBIBP-CorV), Zhifei Longcom recombinant protein vaccine (ZF2001), protein subunits vaccines, and so on. Nowadays, there is a lack of sufficient evidence about the VE for the Omicron variant, but BNT162b2 and mRNA-1273 mRNA vaccines are proved to be effective for the Beta variant that is more likely to cause immune evasion (146). Therefore, current vaccines may be efficacious for the new emergence of the Omicron variant, and there is a need to require a large number of studies to verify the VE against the Omicron strain.

**Table 2:** The best clinical and therapeutic approaches for COVID-19.

**Therapeutic type**	**Drugs**	**Severity of illness**	**Suitable patients**	**Effect**	**References**
Antiviral therapies	Remdesivir	Mild-to-severe	Adults and children (age ≥12 years, weight ≥ 40 kg)	Little or no effect	(130–133)
Anti-SARS-CoV-2 neutralizing antibody products	Convalescent plasma	Severe	life-threatening COVID-19	Effective: Alpha variant; Resistance: Beta variant	(134–137)
	REGN-COV2	Mild-to-moderate	Non hospitalized patients with COVID-19 (age ≥12 years, weight ≥ 40 kg)	Reduced viral load and 70% hospitalization or death rate	(138)
	Bamlanivimab and Etesevimab (LY-CoV555 or LY3819253 and LY-CoV016 or LY3832479)	Mild-to-moderate	Adults	Reduced viral load and 87% hospitalization or death rate	(134, 139)
	Sotrovimab (VIR-7831)	Mild-to-moderate	High-risk non hospitalized patients	Reduced 85% hospitalization or death risk	(140)
Immunomodulatory agents	Corticosteroids	NA	Hospitalized patients without age limitation; pregnant or breast-feeding women	Lower 28-day mortality for patients receiving invasive mechanical ventilation or oxygen support	(141)
JAK inhibitors	Baricitinib	NA	Hospitalized adult patients	Improved the clinical symptoms; reduced 2-week mortality rate and recovery time	(142)
	Ruxolitinib	Severe	Adults (age range: 18–75 years)	The improvement of chest C.T. and faster recovery from lymphopenia	(143)
	Tofacitinib	NA	Adults	Reduced the risk of respiratory failure or death	(144)
	BTK inhibitors (acalabrutinib)	Severe	Adults (age range: 45–84 years)	Improved oxygenation; normalized the CRP, IL-6 and lymphopenia	(145)

*JAK, Janus kinase; BTK, Bruton's tyrosine kinase; CRP, C-reactive protein*.

However, an article showed that anti-RBD neutralizing antibodies against the K417N/E484K/N501Y mutations were inclined to reduce antibodies neutralization, in which E484K accounted for the main cause (147). The E484K mutation participates in immune evasion and is responsible for reduced titers (134). Several lines of evidence implied that neutralization of antibodies induced by two doses of vaccines is potent for most of the variants (59, 148, 149), including the current expanding Delta variant (99). For healthcare workers, a reduction in the occurrence of COVID-19 disease was observed after completing the vaccination (150).

Besides, considering that the elderly are a fragile population, more than 40% of the death of COVID-19 infected victims were elderly people in a sanatorium or other long-term nursing facilities in the United States. At the same time, breakthrough infections often appear in the population aged more than 60 years old. Recent studies based on senior citizens over 80 years old, discovered that the immune response of the elderly was vulnerable but still neutralized VOCs after two doses of vaccines (151). A study suggested that over 75-year-old adults receiving one or two doses of vaccines can resist viral infections, reducing COVID-19-related death cases in Brazil (152). As a result, it is necessary to take effective measures to boost their immune reactions.

However, children have become the dominant affected population by COVID-19 in the United States, and up to September 16, 2021, a total of 5,518,815 children infected with SARS-CoV-2 were reported, among which a proportion of 15.7% of children was infected with COVID-19 [available from: Children and COVID-19: State-Level Data Report (aap.org)]. However, from the perspective of the long run, it is unclear about the impact of the COVID-19 pandemic on children, and viruses may injure the health and influence the mood and the mental status of the affected children. Based on the CDC data, from late June to mid-August 2021, the hospitalization rates of children and adolescents related to COVID-19 caused by the Delta variant increased approximately five times. Meanwhile, the hospitalization rates among unvaccinated adolescents were ten-fold than those fully vaccinated (153). Thus, some clinical doctors urge children who are >12 years old and adolescents who are 12–17 years old to be inoculated with vaccines to fight against COVID-19 illness.

Remarkably, in the light of CDC documents, disease incidence, hospitalization, and death incidence of COVID-19 markedly reduced among the vaccinated population than the unvaccinated population. Furthermore, VE against symptomatic diseases and hospitalization due to infection by the Delta variant were 88% (99) and 96% (154), respectively. Consequently, there is no doubt that vaccination among the public is impending. Also, based on the newest research, the VE against the Delta variant was more than 90%, demonstrating that current vaccines are still potent for the prevailing Delta variant. This threw light on the fact that if there exists sufficient evidence to introduce boosters, it is appropriate for some special populations (155). Table 3 summarizes the basic clinical characteristics of current vaccines, including vaccine type, antigens of the vaccines, target variants, suitable population, efficacy, serious adverse events (SAEs), the immune type generated by bodies, and the phase of trials.

**Table 3:** The basic clinical characteristics of current vaccines.

**Vaccines**	**Target variants**	**Antigens**	**Population**	**Efficacy**	**SAEs**	**Immune type**	**Phase**
Pfizer/BioNTech mRNA vaccine	Alpha, Alpha with E484K, Beta, B.1.526, B.1.617, Gamma, Delta, Delta plus, Lambda and B.1.1.519 lineages	Full-length S protein	Adults; children (5–11 years old); pregnant women	95% (156); 89.5–93.7% (Alpha) (99, 157); 75–100% (Beta) (59, 158); 52.4–88% (Delta) (99, 159); 22.5% (Omicron);89–96% (for pregnant women) (160)	Less than 2%	Humoral immunity	Phase 3 trial (adults); Phase 2/3 trial (children)
Moderna vaccine	SARS-CoV-2 or variants	Segments of SARS-CoV-2 hereditary material	Adults	94.1% (161); 96.4% (Beta) (162); 89% (Alpha); 85% (Beta, Gamma, B.1.617) (146); 50.6% (Delta) (159)	2.9% (one dose)−15.8% (two dose);	Humoral and cellular immunity	Phase 3 trial
AstraZeneca-Oxford vaccine	SARS-CoV-2 or variants	Whole-length S protein	Adults	62.1–79% (78, 163); 61.1% (Beta); 10.4% (Beta; HIV-negative) (164); 74.5% (Alpha); 67% (Delta) (99)	More thrombotic diseases	Cellular immunity	Phase 3 trial
ChAdOx1 nCoV-19/ BNT162b2 mRNA prime-boost vaccination	SARS-CoV-2	Full-length S protein	Adults	91.6% (165)	No	Cellular and humoral immunity	Phase 2 trial
CoronaVac vaccine	Ancestral strains, D614G strains, Alpha, B.1.429, B.1.526, B1.351 and Gamma variants	Inactivated whole SARS-CoV-2 virus	Adults; the elderly aged ≥70 years; healthcare workers	83.5% (166); 50.65% (Brazil); 91.25% (Turkey) (167); 75–88.1% (Alpha), 64.2–70% (Beta), 88.1% (Gamma), 48.33–78.6% (Delta) (seropositivity rate) (168, 169)	No	Humoral immunity	Phase 3 trial
DNA vaccines	G614, Alpha, Beta variants	Plasmid DNA carrying spike-S gene of SARS-CoV-2 virus	Adults	33.33–100% (seroconversion rate) (170)	No	Humoral and cellular immunity	Phase 1/2 trial
NVX CoV-2373 vaccine	Beta variant	SARS-CoV-2 S protein	Adults	86.3% (Alpha) (171); 49.4% (Beta); 60.1% (HIV-negative) (172); 43% (Beta, Gamma, B.1.617) (146)	Rare	Humoral immunity	NA
BBIBP-CorV vaccine	SARS-CoV-2 viruses	SARS-CoV-2 S protein	Adults; children and adolescents	78.1–79.34% (167, 173); 91–96% for adults (seroconversion rate) (174); 99–100% for children and adolescents (seroconversion rate) (175)	No	Humoral immunity	Phase 1/2 trial
ZF2001 vaccine	SARS-CoV-2 viruses	Dimeric RBD-related protein	Adults	72–97% (seroconversion rate) (176)	0.83%	Humoral and cellular immunity	Phase 1/2 trial
SCB-2019 vaccine	SARS-CoV-2 viruses	S-Trimer protein	Younger adults (aged 18–54 years) and older adults (aged 55–75 years)	86–100% (seroconversion rate) (177)	1.4%	Humoral and cellular immunity	Phase 1 trial
Ad26.COV2.S vaccine	D614G, Beta, P.2 lineage	Whole S protein of SARS-CoV-2	Adults	66.9–76.7% (14 days); 66.1–85.4% (28 days) (178, 179); 72% (Alpha); 57% (Beta, Gamma, B.1.617) (146)	No	Humoral and cellular immunity	Phase 1/2a trial; Phase 3 trial

*SAEs, serious adverse events; NA, not available*.

### Immune Responses for SARS-CoV-2 and Variants

Individuals can benefit from these vaccines generating humoral and cellular immunity.

**Figure 3:**
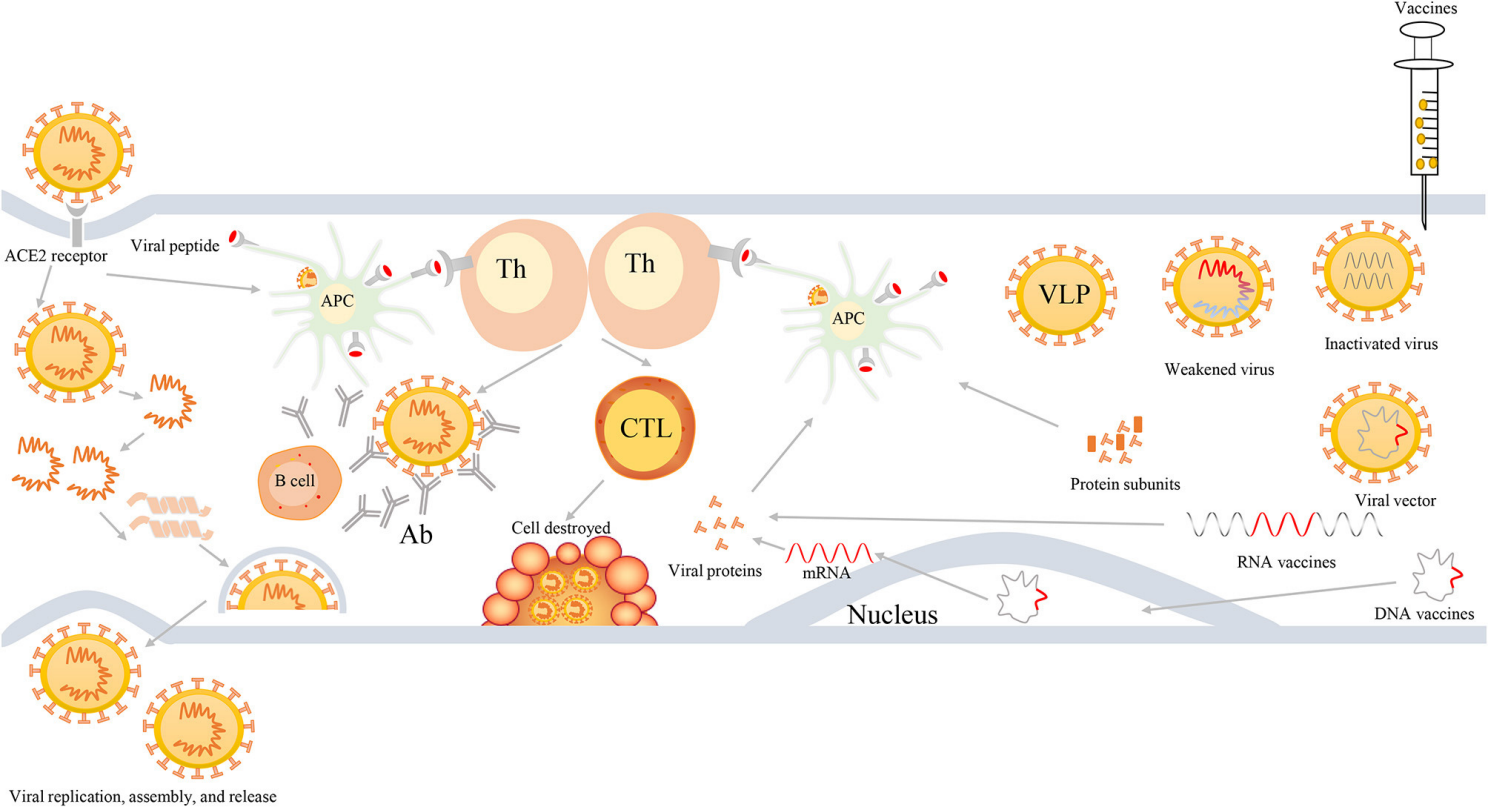
The process of viral infection and induced bodies to generate immune responses after infection or inoculation of vaccines. First, SARS-CoV-2 viruses enter into cells *via* binding to ACE2 receptors, then they release their genetic materials, accomplish replicate, translate RNA into proteins, assemble viruses, and finally release a multitude of viruses. Second, individuals also harbor unique immune mechanism to fight against SARS-CoV-2 infection. When viruses enter into cells, APCs will ingest viruses and initiate immune responses *via* the recognition of viral peptide by MCH molecules. APCs can present the information of viral antigens to Th cells. Subsequently, Th cells are capable of activating humoral immunity to generate antibodies and cell-mediated immunity to lyse and kill viruses. Third, when individuals are inoculated with vaccines, such as weakened or inactivated viruses, viral vector, nucleic acid (DNA or RNA), and protein-based (protein subunits or VLP) vaccines, they can produce effective immune responses to defend infection against SARS-CoV-2 and its variants. Th, T-helper; APC, antigen presenting cells; CTL, cytotoxic T cell; Ab, antibodies; VLP, virus-like particles.

First, viruses enter the cells *via* the viral S protein recognizing and binding to the ACE2 receptor on the host cells. In general, viral RNA itself can act as mRNA, hijack the ribosome of host cells, and thereby complete the process of replication and translation in the host cells. Next, they can produce RNA polymerase and various assembly proteins, making the virus reassemble and release in large quantities. Second, antigen presenting cells (APCs) in the host can usually identify the virus, and present the viral peptides to the T-help cells that are able to stimulate and activate the killer T cells, and then kill the virus-infected cells (cellular immunity). More importantly, viruses are also capable of stimulating the proliferation of B cells, generating neutralizing antibodies, and ultimately eliminating the viruses (humoral immunity). Figure 3 shows the induced immune responses after the infection of viruses or inoculation of vaccines against SARS-CoV-2 or variants.

### Pfizer/BioNTech mRNA Vaccine

The BNT162b2 that belongs to the mRNA vaccine can transcribe and translate the full-length S protein, reaching 95% efficacy against COVID-19 (156). Although the mRNA sequence of BNT162b2 is developed based on the original virus-isolated strains (180), several studies adumbrated that serum-induced by BNT162b2 vaccine still maintained antibodies neutralization against some variants (Figure 3), such as Alpha, Alpha with E484K, Beta, Iota, B.1.617, and Gamma strains (61, 79, 118, 120, 181, 182). Some studies showed that human serum from BTN162b2-immunized individuals was able to effectively neutralize the Delta plus variants (delta AY.1, delta AY.2, and delta Δ144) with modestly reduced neutralization, as well as Lambda and B.1.1.519 variants, and similar neutralization to WT strains (98). In addition, based on a phase 3 placebo-controlled, randomized trial for 44,000 participants, individuals receiving two doses of the BNT162b2 mRNA vaccine can achieve 95% efficacy against SARS-CoV-2 infections (156). It is noted that the potency of BNT162b2 vaccine slightly reduced infection against the Delta variant, with a VE of 88% after completing two doses (99).

As is known, the safety of vaccines is of the greatest concern for the public. Polack et al. reported that <2% SAEs occurred in the recipients of BNT162b2 mRNA vaccine for either dose, apart from fatigue (3.8%) and headache (2.0%) for the second dose (156). Also, according to a study on August 25, 2021, in Israel, the researchers detected the occurrence of myocarditis in nearly 1–5 per 100,000 cases in BNT162b2 mRNA vaccine recipients, but other risks were obviously lower than COVID-19, and they illuminated the potent protection of this mRNA vaccine for healthy population (183).

Nevertheless, due to special circumstances related to pregnant women, most countries regarded them as contraindications of vaccination (184). A study on 10,861 pregnant women receiving BNT162b2 mRNA vaccine in Israel implied that this mRNA vaccine was potent for deterring the SARS-CoV-2 infection with VE of 96% and protecting the participants from severe COVID-19 illness (VE: 89%) (160). Likewise, Shimabukuro et al. conducted a retrospective study on the safety of mRNA vaccines for vaccinating 35,691 pregnant women aged 16–54, and they observed that mRNA vaccines did not increase the risk of adverse pregnancy and the neonatal outcome and mRNA vaccines are safe among the pregnant population (185). Therefore, pregnant individuals are expected to be vaccinated against SARS-CoV-2 and variants in the near future.

To our delight, on September 20, 2021, Pfizer and BioNTech released results from a phase 2/3 trial among 2,268 children aged 5–11 years old after vaccinating two doses of 10 μg BNT162b2 mRNA vaccine, demonstrating that these children can generate sufficient antibodies elicited by 10 μg vaccine, with high neutralization titers, comparable to those produced by individuals of 16–25 years of age vaccinated with 30 μg vaccine. Most importantly, this vaccine was also proven to be of well tolerance and safety for children (available from: Pfizer and BioNTech Announce Positive Topline Results From Pivotal Trial of COVID-19 Vaccine in Children 5 to 11 Years|Pfizer). Consequently, the BNT162b2 vaccine cooperates with the existing public hygiene and health measures, which can help the public to reduce the global catastrophe caused by the expansion of COVID-19. Also, it is urgent to encourage the public to vaccinate with the BNT162b2 vaccine to impede the transmission of current variants and prevent the occurrence of novel variants; then the COVID-19 pandemic is hopeful to be terminated.

### Moderna Vaccine

Moderna mRNA-1273, an mRNA vaccine, utilizes the segments of SARS-CoV-2 hereditary material, to stimulate the body response to the SARS-CoV-2, rather than in a form of weakened or inactivated viruses (Figure 3). The potency of the mRNA-1273 vaccine was proved to be 94.1% for COVID-19 disease, even severe illness, in a phase 3 trial (161), then mRNA-1273 vaccine has been approved for use by the Emergency Use Authorization (EUA). Participants who were vaccinated with two doses of mRNA-1273 vaccine can generate effective antibodies to neutralize SARS-CoV-2 or variants (162, 186–188), but mutations in RBD do reduce the efficacy of antibodies neutralization (47, 55, 189). The latest study elucidated that a low dose (25 μg) mRNA-1273 vaccine can trigger long-lasting antibodies, memory CD4^+^ T cells, including T follicular helper (T_FH_) and IFNγ-expressing cells, and CD8^+^ T cells, reinforcing humoral and cellular immunity (190).

There is no doubt that any vaccine may inevitably generate AEs, and a phase 3 trial also revealed that Grade 3 side effects of the mRNA-1273 vaccine were 2.9% after receiving the first dose and 15.8% after the second dose (161). Besides, the occurrence rate of venous thrombotic events (VTEs) and arterial thrombotic events (ATEs) for Moderna vaccine recipients were 0.075 and 0.13 cases per 1 million individuals, respectively (191). In addition, AEs generated by the mRNA-1273 vaccine were only mild to moderate in the older population (192). Recently, a novel study including more than 6.2 million participants from the Vaccine Safety Datalink that reported the safety of mRNA vaccines, included only either BNT162b2 (Pfizer-BioNTech) or mRNA-1273 (Moderna) vaccines, against SARS-CoV-2, from December 14, 2020 to June 26, 2021. It is reported that the main vaccinated population are young adults aged 18–49, and no serious AEs related to BNT162b2 or mRNA 1273 vaccines were found, except myocarditis/pericarditis occurring in the young population (193), which ease the anxiety and worry about mRNA vaccines against SARS-CoV-2. Thus, it is efficacious and relatively safe to vaccinate mRNA vaccines for the public.

### Oxford-AstraZeneca Vaccine

The Oxford-AstraZeneca vaccine (AZD1222) is a replication-deficient chimpanzee DNA viral vector ChAdOx1 vaccine that possesses the whole-length S protein of SARS-CoV-2 (194), which can assist the immune system in the battle against viral infection (Figure 3). The effectiveness after two doses of AZD1222 was merely 62.1% in a phase 3 trial of 23,000 subjects (78), inferior to BNT162b2 (95%) (156) and mRNA-1273 (94.1%) (161) vaccines. Additionally, AZD1222 is the third approved vaccine by the Medicines and Healthcare Products Regulatory Agency (MHRA) of the UK and other agencies in the world (195). Intriguingly, the interval time of the second dose inoculation is associated with VE. A study found that the effectiveness can reach 82.4% after 12 weeks interval of two doses of vaccines, whereas VE reduced to 54.9% after <6 weeks apart two doses (196).

Nevertheless, the side effects of the Oxford-AstraZeneca vaccine cannot be overlooked, such as thrombotic thrombocytopenia (197–199) that usually occurred in young population, thromboembolism (191, 200), thrombotic adverse (201), skin rash (202) or necrosis (203), psoriasis (204), etc., where most of AEs may ascribe to autoimmune pathological responses after inoculated with vaccines. Madhi et al. (164) performed a multicenter, double-blind, and a randomized controlled trial and they concluded that a two-dose regimen of AZD1222 vaccination cannot provide sufficient protection for mild-to-moderate COVID-19 subjects against the Beta variant infection. Furthermore, a recent study revealed that the efficacy of recipients with two doses of the ChAdOx1 nCoV-19 vaccine only reached 67% against the prevalent Delta variant (99). Therefore, both the efficacy and safety of vaccines deserve to be taken into consideration during the construction of the herd immunity. However, AZD1222 is an ideal vaccine for some resource-limited low- and middle-income nations (205).

Nowadays, a heterologous prime-boost vaccination is emerging, referring to ChAdOx1 nCoV-19 vaccine as prime and BNT162b2 mRNA vaccine as boost vaccination, where combining these two vaccines boost the cellular and humoral immunity to some point. Based on a study of heterologous prime-boost schedule with ChAd/BNT vaccines, researchers found that the schedule owned adequate immunogenicity and was capable of stimulating bodies to generate robust immune responses with a VE of 91.6%, which was higher than ChAd/ChAd vaccine schedule (165). Additionally, in a study on the heterologous vaccination in Germany, the neutralization activity elicited by heterologous regimen (ChAd/BNT) strikingly rose, compared to homologous strategies (ChAd/ChAd or BNT/BNT) (206). Also, in a multicenter, open-label, randomized, phase 2 trial, ChAd/BNT prime-boost scheme significantly elevated the antibodies titers, and no severe AEs were observed in this regimen (207).

### CoronaVac Vaccine

CoronaVac, a chemosynthetic inactivated vaccine, is developed for defending the SARS-CoV-2 strains (208, 209). The CoronaVac vaccine is of safety and well tolerance in the elderly aged more than 60 years and can induce bodies to generate sufficient neutralizing antibodies titers against the COVID-19 illness in phase 1/2 clinical trials (209) (Figure 3). Besides, in a phase 3 trial, among 10,218 volunteers aged 18–59 years old in Turkey from September 14, 2020 to Jan 5, 2021, the VE of CoronaVac was 83.5% (95% CI: 65.4–92.1%) without fatalities or severe AEs (166). Under the circumstance of the Gamma variant expanding, the CoronaVac vaccine, administered to the elderly aged more than 70 years, has decreased the incidence of symptomatic COVID-19, hospitalization, and death cases in Brazil (210).

The efficacy pertinence to the CoronaVac vaccine was rather high (211), and AEs mainly concentrated on mild-to-moderate degree; no overtly serious or life-threatening AEs were observed in Turkey and China (212–214), which laid a foundation for providing highly effective and safe vaccines for the public. More specifically, in a phase 1/2 clinical trial, researchers explored the immunogenicity, tolerability, and safety of the inactivated CoronaVac vaccine in 550 young participants aged 3–17 years in Zanhuang (Hebei, China); then they concluded that CoronaVac vaccine was indeed safe and well-tolerated, capable of inducing humoral responses and generating higher neutralizing antibody titers elicited by 3.0 μg dose, which provides new insight into vaccination using two doses of 3.0 μg regimen for children and adolescents (215). Besides, the mortality rate of healthcare workers who were fully vaccinated with the CoronaVac vaccine declined (216). In addition, circulating neutralizing antibody responses can be markedly boosted among previously seropositive participants after achieving vaccination of two doses of CoronaVac vaccine or one dose of BNT162b2 mRNA vaccine, indicating robust induction of CoronaVac vaccine for memory B cell (217). A single-center study in Ankara, Turkey, revealed that most of the healthcare workers fully immunized using the CoronaVac vaccine accomplished seroconversion, and the younger healthcare workers presented higher IgG levels against SARS-CoV-2 (218).

Current serum elicited by the CoronaVac vaccine can neutralize ancestral strains, D614G strains, Alpha, and Epsilon variants, but reduced neutralization against the Eta and Beta variants (219). Therefore, the CoronaVac vaccine is also a promising choice for the public to vaccinate, and it is necessary for high-risk populations, such as healthcare workers, the elderly as well as individuals with chronic diseases, to perform a booster dose after two doses of vaccines (218).

### DNA Vaccines

DNA vaccines are vaccines directly introduced to the plasmid DNA that encode the immunogen for the acquirement of immune response *in situ* target immunogen (Figure 3). On one hand, stable plasmid DNA is convenient as it can be stored and easily delivered in the setting of room temperature without the cold chain. On the other hand, DNA vaccines are free from forming not only anti-vector immunity but also off-target adaptive immunity to DNA, which is safe for DNA vaccines construction and production (220).

Phase 1 trial of INO-4800 demonstrated that this DNA vaccine was well immunogenic in all 38 vaccinated participants, who generated efficacious humoral and Th1 cell-mediated immunity (221). Furthermore, serum acquired by the INO-4800 vaccine has robust humoral responses against G614 and the Alpha strains but has lower antibodies neutralization against the Beta variants (222). Besides, in phase 1/2 clinical trials *via* intracutaneous injection for healthy Indians, the DNA vaccine (ZyCoV-D) developed by India achieved seroconversion for those who received vaccination and was proved to be safe (170). In consequence, the data support the development of DNA vaccines for defending global public crisis.

### Other Vaccines

NVX CoV-2373 vaccine refers to a recombinant nanoparticle targeting SARS-CoV-2 S protein, where postvaccination serum has the potentiality of neutralizing the Beta variant, with VE of 49.4% (172). BBIBP-CorV and ZF2001 vaccines are developed by China against SARS-CoV-2 infections. BBIBP-CorV endows sufficient productivity and excellent hereditary stability in terms of vaccine manufacturing, which exhibits a promising prospective (223). In addition, a double-blind, randomized phase 1/2 trial illuminated that the BBIBP-CorV vaccine that was well-tolerated and safe, enabled bodies to generate immunized antibodies against SARS-CoV-2 strains (174). Xia et al. evaluated the immunogenicity and safety of the BBIBP-CorV vaccine in children and adolescents aged 3–17 years in a phase 1/2 trial and delineated that the inactivated BBIBP-CorV vaccine was able to induce potent humoral immunity for defending COVID-19 illness, with safety and tolerance (175). Regarding the ZF2001 vaccine, it is a reconstructed dimeric RBD-related protein vaccine that is at phase 3 trial and has been approved for emergency use in China and Uzbekistan (176, 224). A phase 1/2 trial elucidated that the ZF2001 protein vaccine was characterized by immunogenic and easily tolerated traits, and this vaccine was able to induce moderate levels of cellular immunity and potent humoral immunity (176). Furthermore, another protein subunit vaccine, called SCB-2019 vaccine that is composed of S-Trimer protein, can trigger strong humoral and cell-mediated immunity for defending SARS-CoV-2, based on a phase 1, double-blind, randomization trial (177).

Adenovirus type 26 vectors (Ad26.COV2.S, Janssen), a recombinant but replication-incompetent Ad26, encode the whole S protein of SARS-CoV-2. A multicenter, phase 1/2a trial indicated that stable antibodies titers still existed and robust T-cell responses were observed in subjects inoculated with one (cohort 1, aged 18–55 years) or two (cohort 3, aged ≥ 65 years) doses of Ad26.COV2.S vaccine (225). Also, FDA reported a multi-national double-blind, phase 3 randomized trial for 40,000 adult participants receiving Ad26.COV2.S vaccine, which emphasized that VE were 66.9% (95%CI: 59.0–73.4%) for onset at ≥14 days after the admission of one dose vaccine and 66.1% (95%CI: 55.0–74.8%) for the onset at ≥28 days after vaccination (178). Likewise, Sadoff et al. conducted an international, double-blind RCT, and they confirmed that Ad26.COV2.S shielded participants from moderate to severe COVID-19 illness, and VEs against critical COVID-19 disease were higher at least 14 days (76.7%) and 28 days (85.4%) after vaccination (179).

## Conclusions and Recommendations

Over the past 2 years, the COVID-19 pandemic has been ongoing. With the expansion of SARS-CoV-2, novel variants are emerging. A broad array of variants was extensively identified in the United Kingdom, South Africa, Brazil, India, and Peru, based on their respective characterizes, containing higher infectivity, immune escape, increased severity of illness, and hospitalization. Recently, the Delta variant that arouses the greatest concern of the public has been prevailing worldwide, with more contagious and rapidly spreading characteristics. However, numerous studies denoted that current vaccines still have the tremendous potentiality of preventing SARS-CoV-2 and variants, including the Delta variant. Nevertheless, on November 26, 2021, with the appearance of the Omicron variant, there is a growing concern about its transmissibility, virulence, infectivity, immune responses, etc. Currently, it remains unclear whether the Omicron variant will become as prevailing as the Delta variant. Generally, three tricks are utilized by SARS-CoV-2 viruses to enhance viral capability to spread, such as ameliorating the process of gateway to the host's cells, enriching the number of microbes in the host's body for spreading more viruses by breathing, coughing or talking, and facilitating the longer survival of the virus in the non-host environment (167).

In reality, at present, there is a paucity of curative treatments to cure COVID-19 illness. Nevertheless, there are several measures to prevent the public from the infection of SARS-CoV-2 and its variants. First, it is necessary to avoid short-distance contact, keep a certain social distance, reduce social activities, wear surgical or N95 facial masks, and cordon communities where positive cases live or visit when the potentially fast extensive spreading of COVID-19 appears. Second, massive citywide viral RNA screening tests were also essential once the local outbreak of COVID-19 occurred for recognizing unidentified potential cases. Third, the development of big data for real-time surveillance and the management of close contacts with confirmed COVID-19 cases is conducive to epidemiological investigation. Last but not the least, the public also needs immune responses elicited by vaccines to fight against current SARS-CoV-2 and various variants. In spite of the emergence of variants accompanying a multitude of mutations, including E484K, K417N, N501Y, P681R, L452R, T478K, etc., taking positive and effective measures and accomplishment of two doses of vaccination will help prevent the public from the infection against SARS-CoV-2 and its variants, where the vaccines can induce abundant humoral or cellular immune responses and are also potent against its variants to some extent. As a consequence, it is extremely urgent to encourage the public to be inoculated with two doses of vaccines, especially the special populations, such as the elderly, pregnant women, adolescents, and children over 12 years old, if the policy allows. Nowadays, vaccines approved or currently available are effective tools to protect the population from the COVID-19, and vaccination for as many individuals as possible should be given more importance by the administration and authority all over the world. Although the VEs will decrease with the prolonged vaccination time, the severe cases and deaths of COVID-19 patients vaccinated before infection will significantly reduce. Besides, it is necessary to vaccinate the public with boosters.

Mahmud et al. (167) used a mathematical model to investigate the pandemic scenario in California and the entire United States from the day of starting the vaccination program, and they observed that wave peaks will decrease as time goes by, and the epidemic also will be controlled by the middle of 2023. At the same time, the total fatality and recovery rates of the SARS-CoV-2 pandemic in California will be 1.697 and 98.30%, respectively. This phenomenon shows that effective vaccination will prevent and control the SARS-CoV-2 pandemic by developing immunity against viruses.

Moreover, with more and more understanding of the pathological mechanisms of SARS-CoV-2 and variants, and the development of powerful vaccine regimens, the formal guidelines will be more refined. Consequently, we are convinced that the fighting will be a victory and the COVID-19 will be conquered in the future *via* joint effort worldwide.

## Author Contributions

YJ and CY designed and conceived the study. YJ and QW performed literature research and drew the figures and tables. YJ drafted the manuscript. CY and PS critically revised the manuscript. All authors read and approved the final manuscript.

## Funding

This work was supported by the Cuiying Scientific and Technological Program of Lanzhou University Second Hospital (CY2018-MS10) and the project of Lanzhou Science and Technology Bureau (2019-ZD-67).

## Conflict of Interest

The authors declare that the research was conducted in the absence of any commercial or financial relationships that could be construed as a potential conflict of interest.

## Publisher's Note

All claims expressed in this article are solely those of the authors and do not necessarily represent those of their affiliated organizations, or those of the publisher, the editors and the reviewers. Any product that may be evaluated in this article, or claim that may be made by its manufacturer, is not guaranteed or endorsed by the publisher.
